# Vision-Based Topology-Consistent Structural Parsing of Hand-Drawn Circuit Diagrams

**DOI:** 10.3390/s26113440

**Published:** 2026-05-29

**Authors:** Haoyu Wang, Yuhan Wu, Xiaoming Liu, Wen Li

**Affiliations:** 1 School of Electronic Engineering, Nanjing Forestry University, No. 159 Longpan Road, Nanjing 210037, Chinawuyhan@njfu.edu.cn (Y.W.); 2 Postdoctoral Scientific Research Workstation, Nantong Youxing Wiring Harness Co., Ltd., Nantong 226000, China; 3 School of Computer Science, Nanjing Audit University, No. 86 Yushan West Road, Nanjing 211815, China

**Keywords:** hand-drawn circuit diagrams, vision-based sensing, sensing and imaging, camera-acquired images, image acquisition and processing, structural parsing, topology recovery, circuit digitization, SPICE netlist generation

## Abstract

**Highlights:**

**What are the main findings?**

- The proposed framework achieves a 95.14% strict end-to-end success rate on an independent benchmark of 1317 hand-drawn circuit images.
- By integrating multi-source visual perception, wire connected-component-guided reasoning, and terminal semantic recovery, the framework robustly reconstructs circuit topology from noisy camera-acquired hand-drawn inputs.

**What are the implications of the main findings?**

- The study demonstrates a practical vision-based sensing and imaging solution for converting camera-acquired hand-drawn circuit images into SPICE-compatible structural representations.
- The generated SPICE-compatible netlists support downstream ngspice validation, which may benefit circuit education, document digitization, and engineering image-analysis workflows.

**Abstract:**

Hand-drawn circuit diagrams remain common in education, maintenance, and early-stage design and are often photographed for storage, sharing, and reuse. Recovering electrically meaningful structure from such camera-acquired images is difficult because irregular strokes, wire discontinuities, crossings, symbol–text interference, and imaging artifacts can disrupt valid circuit topology. We therefore formulate the task as topology recovery with semantic completion rather than symbol recognition alone. To solve it, we propose a topology-consistent structural parsing framework that integrates multi-source visual perception, wire connected-component-guided connectivity reasoning, and explicit endpoint semantic recovery for direction-sensitive and multi-terminal components. On an independent benchmark of 1317 hand-drawn circuit diagrams, the proposed method achieves a 95.14% strict image-level end-to-end success rate. The recovered structures are further exported as Simulation Program with Integrated Circuit Emphasis (SPICE)-compatible netlists for downstream simulation and verification. These results support a practical vision-based image acquisition and processing workflow for converting camera-acquired hand-drawn circuit images into machine-readable and simulation-ready circuit representations.

## 1. Introduction

### 1.1. Practical Context and Task Scope

Hand-drawn circuit diagrams remain an important medium for technical communication in early-stage design, education, maintenance, and rapid prototyping, where flexible expression and low-cost iteration are often required [1,2,3]. In practice, these sketches are frequently photographed before being stored, shared, or reused. Recovering their electrical meaning from acquired images is therefore valuable not only for archival digitization, but also for circuit simulation, documentation reuse, and integration with electronic design automation (EDA) workflows. The practical goal is not merely to detect visual symbols, but to convert camera-acquired hand-drawn circuit images into machine-readable and simulation-ready circuit representations.

When hand-drawn schematics are acquired under practical conditions, the images are affected by both natural hand-drawing variability and acquisition-stage degradation, including illumination variation, blur, paper texture, cast shadows, perspective distortion, and resolution changes. In such cases, the central difficulty is not symbol recognition alone, but recovery of an electrically valid topology from noisy and sometimes contradictory visual evidence. Real drawings often contain irregular strokes, wire breaks, dense crossings, ambiguous junctions, symbol–text interference, and large style variation, all of which can disrupt circuit connectivity even when individual components are visually recognizable. Related *Sensors* studies on document images and optical character recognition (OCR) under challenging capture conditions likewise show that downstream understanding becomes highly sensitive to non-uniform illumination, blur, and shadow contamination, motivating robust image processing and recognition under realistic acquisition settings [4,5,6]. For hand-drawn circuits, this means that topology recovery—especially wire continuity, crossover handling, junction disambiguation, and terminal-role assignment—becomes the decisive bottleneck for reliable end-to-end parsing.

From this perspective, the task belongs naturally to vision-based sensing and imaging rather than to isolated symbol classification. The input is a camera-acquired technical image, the measurements are imperfect because of realistic acquisition effects, and the desired output is an electrically meaningful structure that remains usable for downstream analysis. More broadly, deep learning has become an effective tool for extracting structured information from sensing data and for accelerating sensor-oriented inverse design in other domains, including 3D plant phenotyping and terahertz biosensor development [7,8,9]. This broader sensing context supports the use of learned perception and optimization modules for converting imperfect measurements into structured outputs, while the present study focuses specifically on circuit-diagram topology recovery. Recent reviews of technical-diagram understanding and visually rich document analysis likewise emphasize that reliable extraction from acquired images requires joint reasoning over symbols, text, layout, and structure rather than isolated object detection alone [10,11]. In the present study, this framing is not only conceptual; robustness is later examined across different diagram-origin groups within the benchmark, and the recovered structures are further tested through downstream SPICE/ngspice execution.

### 1.2. From Symbol-Level Perception to Structural Parsing

Research on hand-drawn circuit understanding has progressed from classical image-processing and pattern-recognition pipelines to more recent deep learning systems for symbol detection and classification [12,13,14,15]. Early methods mainly relied on handcrafted features, stroke analysis, and geometry-driven rules for sketch understanding and isolated symbol recognition [16,17]. These approaches can work in constrained settings, but their robustness degrades when drawing styles vary or strokes are discontinuous and noisy. More recent convolutional, YOLO-based, and transformer-based approaches have substantially improved component recognition performance in hand-drawn circuit images [12,18,19,20], and public datasets such as JUHCCR-v1 have made module-level benchmarking more systematic [21]. However, stronger symbol-level perception does not by itself guarantee a usable circuit graph.

Beyond component detection, several studies have attempted to infer structure through stroke grouping, line tracking, node recognition, graph assembly, and machine-readable reconstruction [14,16,18,19,22,23,24]. Recent modular graph-extraction work on handwritten circuit diagram images likewise underscores the practical importance of integrating object detection, OCR, graph assembly, and rectification within an end-to-end pipeline [25]. For specialized domains such as power electronic converters, integrated pipelines have shown that component recognition, connectivity inference, and simulation can be linked effectively in practice [22]. Related *Sensors* studies on hand-drawn engineering sketches and camera-acquired structural schematics further indicate that robust structure recovery is essential when engineering drawings are processed under imperfect imaging conditions [26,27]. Even so, existing systems are increasingly strong at symbol-level perception, but still struggle to recover electrically valid topology under realistic acquisition degradation, and they rarely treat endpoint semantics as an explicit structural output inside a general hand-drawn parsing pipeline.

Recent progress in vision–language and graph-learning methods also provides an important broader context for circuit understanding. Multimodal large-language-model approaches have begun to address circuit-related visual generation and reasoning tasks; for example, EEschematic uses a multimodal LLM agent to translate SPICE netlists into human-editable analog schematic diagrams [28]. This direction is complementary to the present task: it starts from an already available textual or symbolic circuit description, whereas the present work starts from a camera-acquired hand-drawn raster image and recovers the missing circuit graph, terminal semantics, and SPICE-compatible representation. In parallel, circuit graph neural networks have shown that circuit graphs can be powerful substrates for design automation, topology generation, device sizing, and circuit-property prediction [29,30]. These studies assume or generate circuit graphs as structured objects, while the present work addresses the preceding perception-to-graph problem in which the graph must first be recovered from noisy visual evidence. Thus, VLM/MLLM and GNN methods are closely related at the level of circuit understanding, but they currently address different stages of the circuit digitization and design pipeline.

This remaining gap has three closely related aspects. First, many methods treat component terminals primarily as geometric points, focus on orientation or coarse connection recovery, and do not explicitly model endpoint semantics for direction-sensitive or multi-terminal devices [18,19,22,23,24]. To the best of our knowledge, no prior publicly reported system for general hand-drawn circuit diagram parsing has implemented endpoint semantic recovery as an explicit prediction target within an integrated structural parsing pipeline [15]. Second, connectivity recovery is still often dominated by local geometric rules or post-processing heuristics, making it vulnerable to wire breaks, overlap, distortion, and junction ambiguity [18,19]. Although learned graph models provide a promising direction for edge scoring and graph refinement once reliable candidate nodes and terminals are available [29,30], they do not remove the need for a robust perception front end that extracts candidate components, terminals, text, and wire evidence from degraded hand-drawn images. Third, simulation-oriented end-to-end pipelines have mainly been reported for restricted domains such as power converters rather than for general hand-drawn circuit diagrams [22]. Taken together, these observations suggest that the core research problem should be formulated not as symbol recognition alone, but as topology recovery with semantic completion under realistic image-acquisition degradation.

### 1.3. Problem Formulation, Contributions, and Paper Organization

Accordingly, we formulate the task as *topology recovery with semantic completion*. To solve this task, we propose a *topology-consistent structural parsing framework* for camera-acquired hand-drawn circuit images. The framework integrates multi-source visual perception, wire enhancement, wire connected-component (CC)-guided connectivity reasoning, fine-grained component refinement, endpoint semantic recovery, and structured fusion to produce electrically meaningful circuit graphs. The recovered structures can be exported as Simulation Program with Integrated Circuit Emphasis (SPICE)-compatible netlists and further examined by downstream ngspice execution. This design directly targets the main bottleneck identified above: reliable topology recovery from imperfect visual evidence, rather than component recognition in isolation.

The main contributions of this study are as follows:

- We propose a topology-consistent structural parsing framework in which *wire CC-guided connectivity reasoning* is the core methodological module rather than an auxiliary post-processing step. Instead of relying on nearest-neighbor linking alone, the method combines light wire repair, point-to-wire snapping, explicit straight-through crossover handling, within-CC sparse graph selection, and short-range inter-CC bridge validation to recover electrically valid topology from noisy hand-drawn evidence.
- We introduce, to the best of our knowledge, the first explicitly modeled *endpoint semantic recovery* module reported for general hand-drawn circuit diagram parsing, enabling terminal-role inference for direction-sensitive and multi-terminal devices and making the recovered structure electrically interpretable rather than merely geometrically connected.
- We establish a unified end-to-end workflow from *camera-acquired* hand-drawn circuit images to machine-readable and SPICE-compatible circuit representations, and evaluate it on a strictly independent 1317-image benchmark using a strict image-level criterion together with subgroup robustness analysis, downstream ngspice validation, and aligned public-subtask references on JUHCCR-v1 and CGHD [21,31].

On the independent benchmark, the proposed method achieves a 95.14% strict image-level end-to-end success rate and a 96.89% connectivity inference accuracy. Taken together, these results support the effectiveness of a connectivity-centered formulation of topology recovery with semantic completion under realistic hand-drawn and image-acquisition variability, and they provide an evidence chain linking image acquisition, structure recovery, and executable circuit representation.

## 2. Materials and Methods

### 2.1. Framework Overview and Problem Definition

This study addresses end-to-end structural parsing of camera-acquired images of hand-drawn circuit diagrams. More specifically, we formulate the task as *topology recovery with semantic completion*: given an acquired circuit image, the system should recover not only component identities, but also text-linked attributes, electrically valid connectivity, and terminal-role semantics needed for downstream interpretation. Framed this way, the problem is not limited to isolated symbol recognition; it is a vision-based image-acquisition and image-processing problem in which structurally meaningful circuit recovery must remain robust to realistic hand-drawing variability and acquisition degradation.

To solve this task, we implement a fixed ten-stage *topology-consistent structural parsing framework*: **(1) multi-class object detection**, **(2) text recognition and text–component association**, **(3) keypoint detection**, **(4) keypoint aggregation and stabilization**, **(5) wire-structure enhancement**, **(6) wire connected-component (CC)-guided connectivity reasoning**, **(7) endpoint semantic inference**, **(8) multi-source information fusion**, **(9) SPICE netlist generation**, and **(10) downstream circuit simulation**. All reported experiments use this fixed execution order.

**Figure 1:**
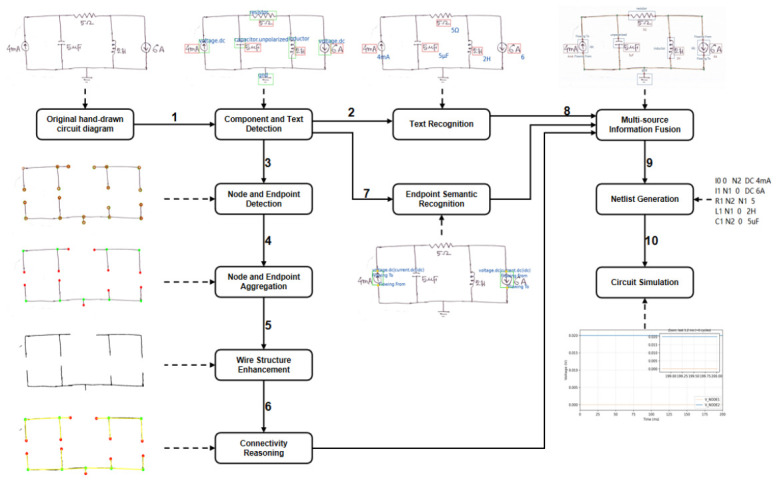
Overview of the proposed framework, illustrated with a coupled resistor–inductor–capacitor (RLC) circuit example. Colors, numbers, and arrows indicate stage grouping, execution order, and information flow, respectively.

Figure 1 summarizes the full processing chain from acquired hand-drawn input to structured output. The framework combines local visual perception of components, text, and terminals with wire-supported topology recovery and endpoint-semantic completion so that the final representation remains directly exportable to SPICE-compatible netlists. Algorithm 1 condenses the fixed execution order used throughout the study and shows how the released system moves from acquired image evidence to structured circuit output and downstream simulator-ready export.

**Figure 2:**
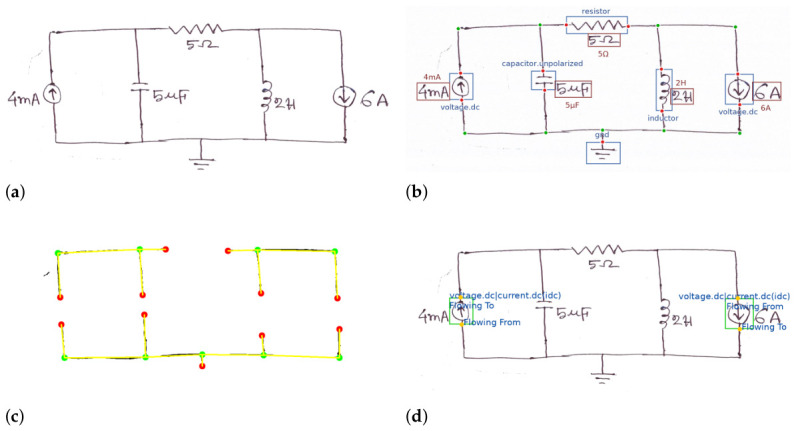
Enlarged intermediate outputs for the coupled RLC example in Figure 1. (**a**) Input hand-drawn circuit. (**b**) Local visual perception result. (**c**) Wire connected-component (CC)-guided connectivity reasoning. (**d**) Endpoint semantic recognition. Colors and arrows indicate detected regions, inferred connections, and processing flow.

**Figure 3:**
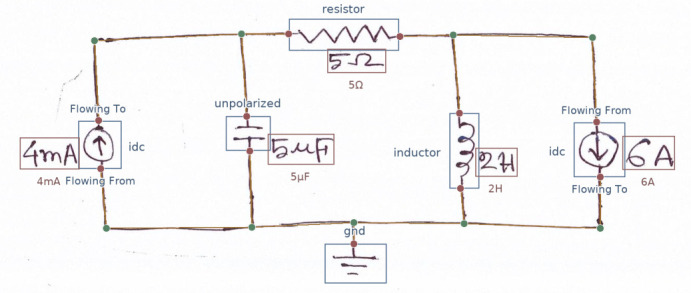
Final fused structured parsing result for the coupled RLC example in Figure 1. Colored circles indicate recovered nodes or connection points in the fused circuit structure.

To concretely illustrate the stage-wise behavior of the proposed framework, the coupled RLC circuit in Figure 1, Figure 2 and Figure 3 is used as an illustrative workflow example. Figure 2 highlights representative intermediate outputs from local perception, wire CC-guided connectivity reasoning, and endpoint semantic inference, while Figure 3 shows the fused structured result that is subsequently exported for SPICE netlist generation and downstream simulation. This example is included for methodological illustration of the processing chain, not as quantitative benchmark evidence.
**Algorithm 1:** Top-Level Pipeline for Topology-Consistent Structural Parsing.     **Input**: Camera-acquired hand-drawn circuit image *I*     **Output**: Structured circuit graph G and SPICE netlist S **1**   D← detect components, text, terminals, and crossovers in *I*  **2**   $A_{\mathrm{text}}\leftarrow$ recognize cropped text and associate it to nearby components using an adaptive scale-aware rule **3**   P← predict candidate wire-junction and component-terminal keypoints **4**   (N,T)← aggregate and stabilize keypoints into circuit nodes and component terminals **5**   W← suppress component interiors and enhance wire structure in *I* **6**   E← recover electrical connectivity by wire CC-guided reasoning on (N∪T,W) **7**   $A_{\mathrm{sem}}\leftarrow$ infer endpoint semantics for supported direction-sensitive and multi-terminal components **8**   G← fuse detections, text-linked attributes, connectivity, and endpoint semantics into a unified circuit graph **9**   S← export G as a SPICE-compatible netlist **10**   optionally execute S in downstream ngspice validation **11**   **return** G,S


### 2.2. Training Data Sources

Different stages of the framework are trained using task-specific datasets rather than a single shared annotation source. Table 1 summarizes the main training sources, their roles in the pipeline, and the evaluation benchmark used only for final testing.

For component/text detection, the training data were formed by merging the publicly available CGHD dataset [31] and Digitize-HCD dataset [32] with additional hand-drawn circuit samples collected and annotated in this study. The resulting unified label system contains 57 classes, including 54 electronic component classes and three auxiliary non-component classes (*text*, *terminal*, and *crossover*). The OCR dataset was created by cropping text instances from the detection training pool. The node/terminal dataset contains manually annotated wire-junction and component-terminal keypoints together with line annotations for HAWP-based training. The endpoint-semantic dataset was derived from Digitize-HCD terminal-semantic annotations [32] and then manually verified and corrected before training.

### 2.3. Independent Benchmark, Annotation Protocol, and Subgroup Definitions

The independent benchmark used for final evaluation contains 1317 hand-drawn circuit diagrams and was kept fully separate from all training and validation data. All benchmark images were acquired by camera. The benchmark contains two mutually exclusive diagram-origin groups: 972 photographs of pre-existing hand-drawn diagrams and 345 photographs of newly drawn hand-drawn diagrams created by researchers to enrich coverage of common component categories and circuit structures. Across both groups, natural hand-drawing artifacts such as stroke-width variation, symbol distortion, irregular spacing, and layout inconsistency were intentionally retained. Camera-acquisition effects such as illumination variation, paper texture, shadows, and resolution differences were also preserved.

Ground-truth labels were produced under a predefined annotation protocol. Component classes, text strings, circuit nodes, connectivity relations, and endpoint-semantic labels were annotated using consistent rules. Wire crossings and junctions were annotated according to explicit visual cues. Arc- or bridge-style crossings were labeled as non-junction crossover structures, meaning that the wires visually cross but are not electrically connected. In contrast, intersections marked with a junction or solder dot were labeled as electrical junctions, meaning that the incident wires belong to the same circuit node. Thus, visually crossing wires were not automatically treated as electrical connections unless explicit node evidence was present. Text was linked to the owning component for OCR/association evaluation, and terminal-role labels were recorded for supported direction-sensitive or multi-terminal component families. Benchmark annotations were generated and cross-checked by two annotators. The first annotator produced the initial structured annotations, and the second annotator independently reviewed the complete benchmark under the same annotation guideline. To quantify annotation reliability, we report image-level exact inter-annotator agreement, where an image was counted as agreed only when the two annotators produced the same structured annotation, including component labels, text ownership, circuit connectivity, junction/crossover interpretation, and endpoint-semantic labels where applicable. The two annotators reached complete agreement on 1294 of the 1317 benchmark images, corresponding to an initial exact inter-annotator agreement of 98.25%. The remaining 23 images were reviewed case by case, and final labels were assigned only after consensus adjudication. Typical disagreements involved dense crossings, faint strokes, unclear handwritten marks, or component–text ownership in crowded regions. Rule-based consistency checks were applied before final release. The overall composition of the independent benchmark, together with the predefined subgroup definitions used in subsequent analyses, is summarized in Table 2.

These statistics indicate that the benchmark is structurally nontrivial and source-diverse, with frequent crossover patterns, widespread endpoint-semantic applicability, and a broad component-count range. Importantly, the subgroup definitions used later in the Results section were fixed from benchmark composition in advance rather than introduced after inspection of model performance.

### 2.4. Pipeline Modules

The released system was implemented as a fixed staged workflow covering detection, OCR, node/terminal prediction, wire enhancement, connectivity reasoning, endpoint semantic inference, structured fusion, and netlist generation/simulation. Across all reported experiments, the execution order was kept unchanged and the module-specific settings were frozen in the version-locked configuration bundle associated with the manuscript snapshot.

#### 2.4.1. Input Acquisition and Preprocessing

The pipeline operates on RGB or grayscale input images; in the independent benchmark evaluated in this study, all inputs are camera photographs of hand-drawn circuit diagrams. Before inference, each image is resized while preserving aspect ratio so that the longer side equals 1024 pixels, followed by intensity normalization and 8-bit conversion. This preprocessing standardizes computational input scale and intensity range but does not remove genuine acquisition-related degradation; no manual cleaning, stroke repair, or redrawing is performed before inference.

#### 2.4.2. Component and Text Detection

Electronic components, text regions, terminal markers, and crossover markers are jointly detected using a You Only Look Once version 10 (YOLOv10)-based multi-class detector [33]. A unified detection formulation is adopted because handwritten text and component symbols often overlap or appear in close proximity, making a decoupled detection sequence less reliable for downstream parsing. During inference, low-confidence detections are discarded and non-maximum suppression is applied to overlapping predictions. The component taxonomy also accounts for common resistor-symbol conventions: zigzag resistor symbols and rectangular resistor symbols are both mapped to the same semantic class, *resistor*, whereas fuse symbols are retained as a separate component class. Thus, the detector distinguishes supported resistor styles from fuse symbols at the component-detection stage rather than treating all visually rectangular or resistor-like symbols as the same class.

#### 2.4.3. Text Recognition and Text–Component Association

Detected text regions are cropped and recognized using a PARSeq-based OCR model trained on 95,544 cropped text samples [34]. The recognized strings are then associated with nearby detected components to recover labels and handwritten parameter values required for structured circuit representation.

This association step is necessary because hand-drawn circuit text varies markedly in size, placement, and writing style. A fixed global pixel threshold is therefore unreliable: a threshold suitable for a large handwritten value may reject a nearby small identifier, whereas a more permissive threshold may attach unrelated text in dense layouts. The released pipeline instead uses a *nearest-candidate plus adaptive acceptance* rule. For each recognized text instance, the nearest candidate component is first identified by spatial proximity. The match is accepted only when the text–component distance is below a threshold proportional to the text-box scale. In this way, larger text regions are allowed a proportionally larger matching radius, while smaller text regions are constrained more tightly.

Algorithm 2 summarizes this scale-aware association procedure. This simple design improves the stability of text–component matching across different handwriting scales and local drawing densities.
**Algorithm 2:** Adaptive Text–Component Association.
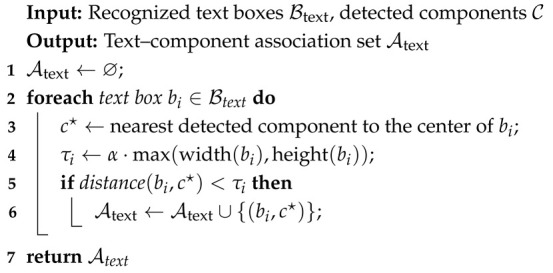
 

#### 2.4.4. Keypoint Prediction, Aggregation, and Stabilization

Candidate wire-junction and component-terminal points are predicted using a Holistically Attracted Wireframe Parsing (HAWP)-based wireframe parsing model [35]. In preliminary experiments, both HAWP and LCNN [36] were trained on the same task. Direct line-level outputs from both models were unstable under hand-drawn conditions because wires are often curved, fragmented, weakly connected, and inconsistent in stroke width. For this reason, the released system does *not* directly trust raw wireframe segments as the final topology substrate. Instead, it retains the more stable HAWP point predictions as candidate circuit keypoints and lets the subsequent wire-enhancement and connectivity stage decide which point-to-point relations are actually supported by wire evidence. This point-first design is important because it separates *where potentially meaningful junctions or terminals are* from *how they should be connected*.

Because multiple nearby predictions may correspond to the same physical junction or terminal, the candidate points are merged using Density-Based Spatial Clustering of Applications with Noise (DBSCAN)-based clustering [37]. Cluster centers are then partitioned into circuit nodes and component terminals according to their spatial relation to detected component boxes. This aggregation and stabilization step reduces duplicate predictions, suppresses jitter from local heatmap offsets, and improves the reliability of the subsequent connectivity stage. Algorithm 3 summarizes the operational logic of this stage.
**Algorithm 3:** Adaptive Keypoint Aggregation and Node/Terminal Partitioning.
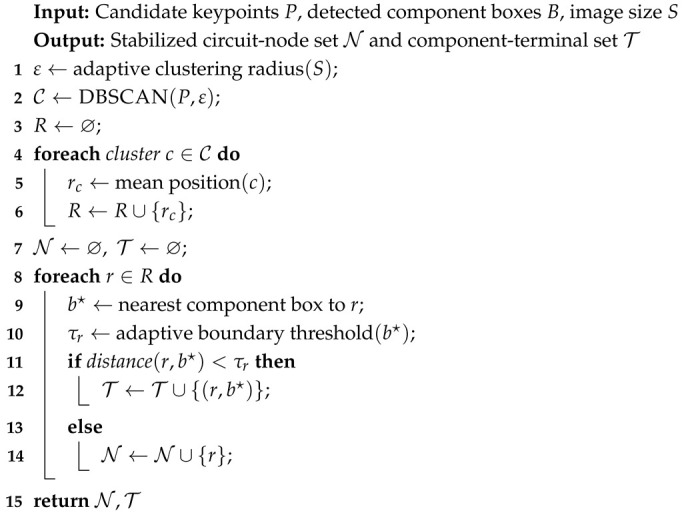
 

#### 2.4.5. Wire Enhancement and Wire CC-Guided Connectivity Reasoning

Detected component regions are suppressed, and the remaining image content is enhanced to recover weak or fragmented wire strokes. The resulting wire mask provides the structural evidence for subsequent connectivity reasoning.

The reasoning procedure contains five components. **(A) Wire CC construction with light repair:** before connected-component labeling, a mild repair step bridges only small local gaps to reduce unnecessary fragmentation of weak strokes. **(B) Point-to-wire snapping:** stabilized circuit nodes and component terminals are snapped to the nearest valid wire pixels so that graph reasoning is anchored to observed wire structure. **(B0) Explicit crossover straight-through pairing:** detected arc- or bridge-style crossover markers are treated as non-junction events, and opposite local directions are paired to preserve straight-through continuity without introducing spurious electrical nodes. By contrast, junction-dot or solder-dot evidence is treated as an electrical node cue, so the incident wires are fused into the same circuit node. **(C) Within-CC sparse graph selection:** candidate edges are first resolved inside each retained wire CC under wire-support and degree constraints. **(D) Short-range inter-CC bridge validation:** only after within-CC reasoning are limited cross-CC repairs considered, and they are accepted only when local dilation and sampled wire support jointly indicate a plausible short bridge.

This design uses wire CC membership as the primary structural prior and applies inter-CC repair only when necessary. Algorithm 4 summarizes the procedure.
**Algorithm 4:** Wire CC-Guided Connectivity Reasoning.
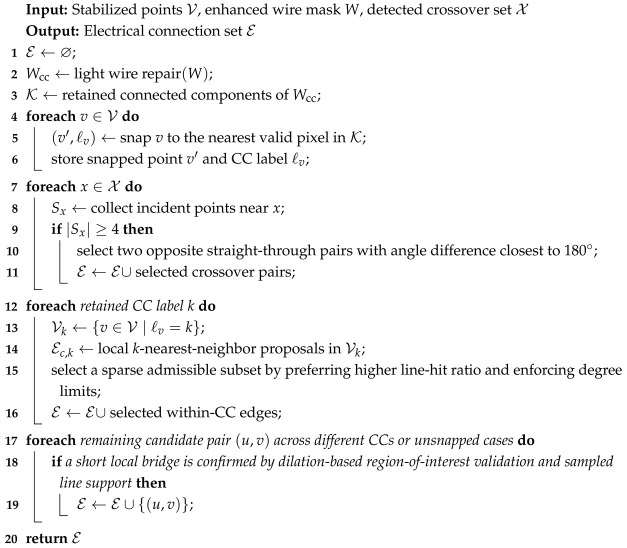
 

#### 2.4.6. Endpoint Semantic Inference, Structured Fusion, and Netlist Export

Direction-sensitive and multi-terminal devices require more than connectivity alone, because their terminal roles affect electrical interpretation. In this sense, endpoint semantics is not an auxiliary embellishment of the graph; it is part of what makes the recovered structure electrically meaningful. Unlike prior systems that typically stop at terminal localization, component orientation, or coarse connection reconstruction [18,19,22,23,24], the released system explicitly performs endpoint semantic inference. Fine-grained subtype refinement is first applied where needed using a DINOv2-based classifier for fine-grained subtype refinement. Endpoint semantics is then obtained in two steps. First, a heatmap-based endpoint predictor, instantiated with a Vision Transformer Pose Estimation (ViTPose)-style top-down architecture, localizes ordered terminal keypoints for supported two-terminal and three-terminal component families [38,39]. Second, component-family-specific semantic mapping assigns electrical terminal roles, such as anode/cathode, gate/drain/source, or base/collector/emitter, using the predicted endpoint order, component subtype, local orientation, and recovered connectivity. The ViTPose-style model is therefore used as a generic keypoint-heatmap estimator trained on circuit-terminal annotations, rather than as a human-skeleton prior. The inferred roles, together with component classes, recognized text, stabilized points, and recovered connectivity, are fused into a unified structured circuit representation.

The final structured representation is exported as a SPICE-compatible netlist for downstream simulation. In the present work, ngspice is used as the simulator-side validation environment [40].

### 2.5. Evaluation Protocol

The primary endpoint was the *strict image-level end-to-end success rate* under a strict image-level binary criterion. A test image was counted as successful only when component classification, connectivity reconstruction, text recognition with text-to-component association, and endpoint semantic inference were all correct. The independent 1317-image benchmark was strictly excluded from model training, validation, hyperparameter tuning, ablation selection, and checkpoint selection throughout all reported experiments.

Secondary outcomes included dimension-specific image-level accuracies for the same four dimensions, evaluated against manually annotated ground truth. For OCR/association and endpoint semantics, applicability coverage and conditional image-level accuracies were additionally reported so that the effective denominators for non-applicable cases were explicit.

To provide a finer-grained evaluation of the connectivity-focused contribution, connectivity was additionally evaluated at the terminal-pair graph level. For each circuit image, the ground-truth and predicted topologies were converted into sets of unordered terminal-pair connectivity relations:

(1) $$E\left(G\right)=\left\{\left\{t_{i},t_{j}\right\}|i<j,\;\mathrm{net}\left(t_{i}\right)=\mathrm{net}\left(t_{j}\right)\right\},$$

where $t_{i}$ and $t_{j}$ denote component-terminal instances and net(·) denotes the electrical net assignment. Terminal-pair precision, recall, and $F_{1}$ were then computed from the aggregated true-positive, false-positive, and false-negative terminal-pair relations:

(2) $$P=\frac{\mathrm{TP}}{\mathrm{TP}+\mathrm{FP}},\;R=\frac{\mathrm{TP}}{\mathrm{TP}+\mathrm{FN}},\;F_{1}=\frac{2PR}{P+R}.$$

This metric is invariant to arbitrary net naming and directly evaluates electrical topology rather than raw wire-pixel overlap or intermediate junction placement. We also report the connectivity edit count, defined as FP+FN, to quantify how many terminal-pair relations would need to be added or removed to match the ground truth.

Downstream utility was assessed from the full 1317-image benchmark through a four-step funnel: SPICE netlist exportability, simulation eligibility, successful ngspice execution, and manually inspected downstream simulation correctness.

For proportion-based results reported with confidence intervals, two-sided 95% CIs were computed using the Wilson score interval [41]. Runtime was measured under single-image inference (batch size = 1) and excluded disk input/output and simulator execution time.

### 2.6. Implementation Environment and Reproducibility

All reported results were generated from the version-locked public repository snapshot associated with this manuscript, together with the released pretrained weights. Table 3 summarizes the main resources needed to recreate the execution environment and run the pipeline.

This version-locked release fixes the code snapshot, execution environment, and pretrained weights used for the reported experiments. Module-specific training details and configuration files are provided in the public repository.

## 3. Results

This section summarizes the end-to-end, graph-level, robustness, ablation, and SPICE executability results on the independent benchmark.

### 3.1. Overall End-to-End Performance

On the independent benchmark, the proposed method achieves a strict image-level end-to-end success rate of 95.14% (1253/1317; 95% confidence interval (CI), 93.84–96.18%, Wilson), as summarized in Table 4.

Under this strict criterion, 64 of the 1317 test images fail on at least one evaluated dimension. The error distribution is uneven across dimensions: component classification, OCR/association, and endpoint semantics all remain above 98% on the all-image denominator, whereas connectivity inference is lower at 96.89% and contributes the largest residual error pool.

To further characterize connectivity quality beyond binary image-level success, Table 5 reports terminal-pair graph-level metrics. The recovered topologies contain 25,158 true-positive terminal-pair relations, 103 false-positive terminal-pair relations, and 50 false-negative terminal-pair relations over the full 1317-image benchmark. This corresponds to 99.59% terminal-pair precision, 99.80% terminal-pair recall, and 99.70% terminal-pair F1. The mean connectivity edit count is 0.12 terminal-pair edits per image, and the median edit count is 0, indicating that the strict image-level failures are concentrated in a small subset of cases rather than reflecting widespread graph corruption.

A residual-error breakdown across the failed cases further supports this pattern. Among the 64 failed images, 41 contain connectivity errors, compared with 23 involving OCR/association errors, 15 involving component-classification errors, and 8 involving endpoint-semantic errors. These error categories overlap and therefore do not sum to the total number of failed images. The image-level and terminal-pair metrics should therefore be interpreted together: the former measures whether an entire circuit is reconstructed without any topological error, whereas the latter distinguishes near-correct graphs from cases with many missing or spurious connectivity relations.

### 3.2. Conditional and Dimension-Wise Results

Because OCR/association and endpoint semantics are not applicable to every image, their conditional results are reported separately in Table 6. Endpoint semantic completion is not a marginal corner case in this benchmark: 1261/1317 images (95.75%) contain endpoint-semantic-sensitive components, and OCR/association is applicable in 1278/1317 images (97.04%).

### 3.3. Endpoint-Semantic Predictor Ablation

To examine whether terminal-role inference can be solved by simple orientation-template mapping, we compared the proposed ViTPose-style heatmap endpoint predictor with two simpler alternatives. The rule-based orientation mapping baseline does not use a trainable model; it assigns terminal roles from the component category, bounding-box geometry, and the relative positions of detected terminals. The lightweight orientation classifier predicts a discrete rotation/mirroring configuration for each component crop and then assigns terminal roles using component-family-specific templates. Thus, this ablation directly tests whether endpoint semantics in camera-acquired hand-drawn diagrams can be recovered by idealized orientation and mirroring rules alone.

As shown in Table 7, the rule-based orientation mapping baseline achieves only 81.76% accuracy, indicating that idealized rotation/mirroring templates are insufficient for many camera-acquired hand-drawn diagrams. The lightweight orientation classifier improves the accuracy to 96.27%, but still produces 47 endpoint-semantic errors. In contrast, the ViTPose-style heatmap endpoint predictor reaches 99.37%, reducing the number of errors from 47 to 8 compared with the lightweight classifier. These results support the use of a heatmap-based endpoint formulation under irregular terminal placement, hand-drawn deformation, and imperfect component localization. At the same time, the comparison shows that ViTPose is not claimed to be uniquely necessary; rather, it is the high-accuracy implementation used in the present pipeline, and lighter endpoint predictors remain promising for resource-constrained deployment.

### 3.4. Robustness Across Diagram Origins and Structural Complexity

**Figure 4:**
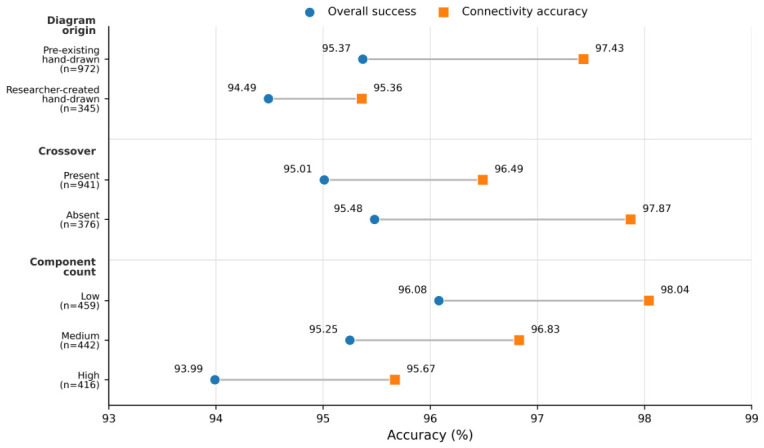
Subgroup results for strict image-level end-to-end success rate and connectivity accuracy across diagram origin, crossover presence, and component-count complexity.

Figure 4 summarizes subgroup performance across diagram origin, presence or absence of crossover structures, and component-count complexity. These subgroup definitions were fixed by benchmark composition rather than being introduced after inspection of the results.

Across diagram-origin groups, the framework achieves 95.37% strict image-level end-to-end success and 97.43% connectivity accuracy on photographs of pre-existing hand-drawn diagrams (927/972 and 947/972, respectively), and 94.49% strict image-level end-to-end success and 95.36% connectivity accuracy on photographs of newly drawn researcher-created diagrams (326/345 and 329/345). These results indicate that high performance is maintained across both source-origin groups rather than being confined to either legacy diagrams or newly created benchmark material.

A similar pattern is observed for structural difficulty. When crossover structures are present, the method reaches 95.01% strict image-level end-to-end success and 96.49% connectivity accuracy (894/941 and 908/941), compared with 95.48% and 97.87% (359/376 and 368/376) on images without crossovers. Across component-count strata, strict image-level end-to-end success decreases from 96.08% in the low-complexity subgroup to 95.25% in the medium-complexity subgroup and 93.99% in the high-complexity subgroup, while connectivity accuracy decreases from 98.04% to 96.83% and 95.67%, respectively. Even in the highest-complexity subgroup, the end-to-end success rate remains above 93%.

### 3.5. Residual Connectivity Error Analysis

To better understand the remaining topology errors, we manually reviewed all 41 test images that failed on connectivity inference and recorded whether each image exhibited one or more recurring local contributing factors. Table 8 summarizes this descriptive analysis. Because the factors are not mutually exclusive, the counts are presence-based rather than single-label assignments, and the corresponding percentages do not sum to 100%.

Weak or thin wire traces are the most frequent contributing factor (22/41, 53.66%), followed by crowded nearby keypoints (17/41, 41.46%) and incomplete component suppression (13/41, 31.71%). In addition, 8 of the 41 connectivity-error images (19.51%) exhibit multiple contributing factors simultaneously.

**Figure 5:**
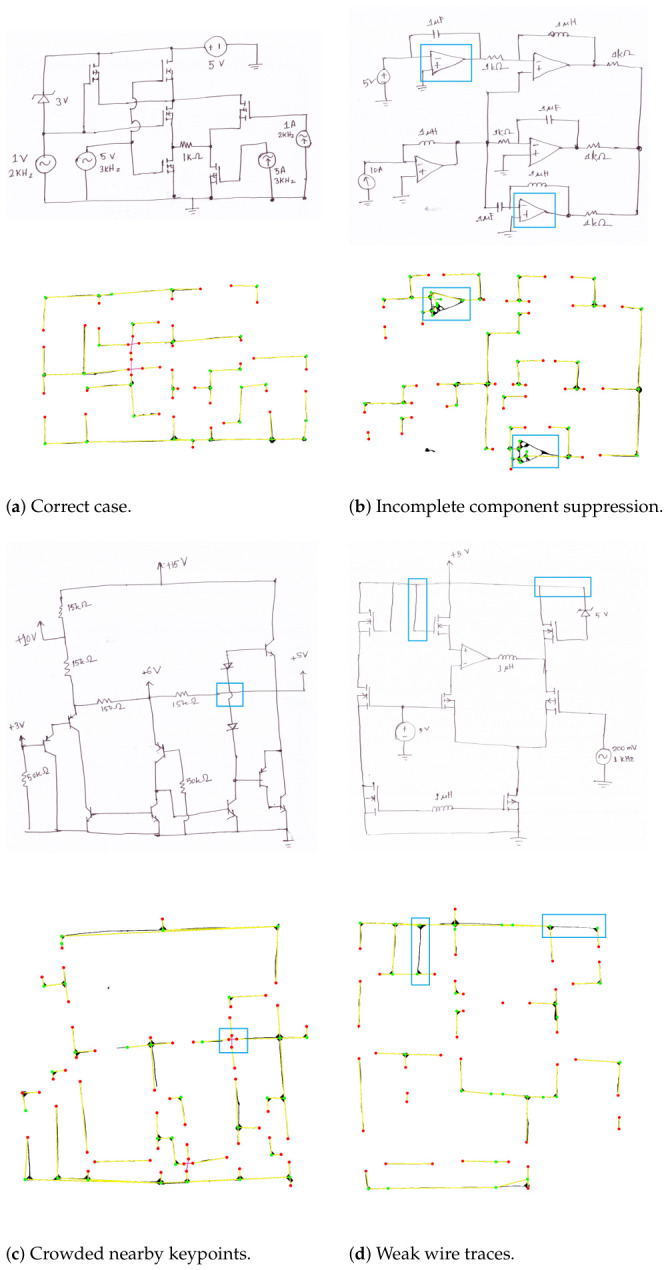
Representative connectivity reasoning case groups. In each group, the top image is the original hand-drawn circuit and the bottom image is the corresponding connectivity-reasoning visualization. Colored overlays indicate connectivity-reasoning outputs, and blue boxes mark the corresponding local regions used to compare the original image with the inferred connectivity result.

Figure 5 provides representative examples of these residual cases. Panel (a) shows a successful example with nontrivial local structure, whereas panels (b)–(d) show representative failures associated with incomplete component suppression, crowded nearby keypoints, and weak wire traces, respectively. Matching blue boxes highlight the erroneous local regions in the connectivity reasoning results and their corresponding regions in the original images, enabling direct comparison between the local visual ambiguity and its topological consequence.

### 3.6. Ablation of the Connectivity Reasoning Module

**Figure 6:**
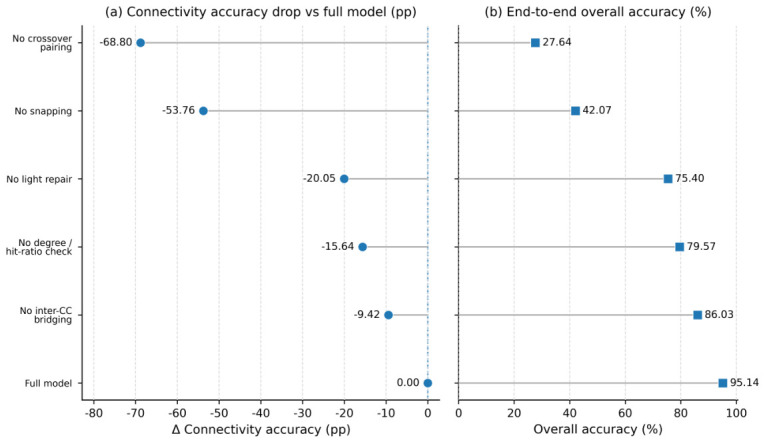
Ablation of the connectivity reasoning module. (**a**) Connectivity-accuracy drop relative to the full model, where the dashed vertical line marks the full-model reference (0 pp). (**b**) End-to-end overall accuracy for each setting.

Because connectivity remains the main bottleneck of the full pipeline, we further examined the contribution of the wire CC-guided reasoning stage. Figure 6 removes one connectivity element at a time while keeping all other stages unchanged, and reports both connectivity accuracy and strict image-level end-to-end accuracy.

All five elements are beneficial, but their effects are clearly unequal. The largest degradations occur when snapping or crossover straight-through pairing is removed. Removing snapping substantially increases broken or false attachments under the primary strict image-level criterion. Removing crossover straight-through pairing causes the most severe degradation: connectivity inference becomes incorrect on 947 of the 1317 test images, and the full pipeline fails on 953 images. The graph-level terminal-pair results in Table 5 complement this ablation by showing that the full connectivity module achieves 99.70% terminal-pair F1 with only 153 total terminal-pair edits over the complete benchmark. Together, these results show that the observed end-to-end performance depends strongly on the explicit topology-reasoning design rather than on recognition modules alone.

### 3.7. Downstream Simulation Validation and Representative Case

**Figure 7:**
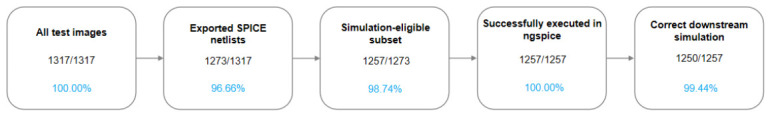
Downstream validation funnel distinguishing SPICE netlist export, simulator eligibility, execution, and result-level simulation correctness.

Figure 7 summarizes the predefined four-stage SPICE validation funnel.

Downstream utility was evaluated using the predefined four-stage SPICE validation funnel. This funnel was designed to distinguish simulator executability from result-level simulation correctness. In this study, a syntactically complete exported netlist indicates that the recovered structure can be represented in SPICE form. A simulation-eligible netlist indicates that ngspice can parse and execute the requested analysis after the required external device models, indispensable component parameters, and analysis commands are available. By contrast, simulation correctness was assessed only after execution, by inspecting whether the exported topology, node mapping, component values, device models, terminal roles, and resulting operating-point values or waveforms were consistent with the source diagram and with the expected behavior of the circuit. Therefore, successful execution was treated as a necessary but not sufficient condition for downstream correctness.

Of the 1317 test images, 1273 were converted automatically into syntactically complete SPICE netlists. Under the baseline simulator environment, 760 exported netlists were immediately simulation-eligible, whereas 513 were initially non-executable because required external device or subcircuit models were unavailable. After the required vendor or public model libraries were supplemented, 497 of those 513 cases were recovered, yielding 1257/1273 exported netlists (98.74%) that were simulation-eligible. The remaining 16 exported but non-simulatable cases were due to missing indispensable handwritten parameters in the original diagrams rather than export failure.

The 1257 executable cases were then examined at the result level. Manual post-execution inspection judged 1250 of the 1257 executed cases (99.44%) to be simulation-correct. A case was counted as correct only when the generated netlist preserved the intended circuit topology and device semantics and when the resulting ngspice output was consistent with the expected circuit behavior. The remaining seven cases executed but were not counted as correct. Among them, five were already counted as end-to-end structured parsing failures, and the other two reflected downstream exporter/simulator-side deviations introduced after successful image-level structural parsing. This distinction shows that the downstream validation did not equate “executable” with “correct”.

Table 9 summarizes the causes of initial non-executability among exported netlists.

**Figure 8:**
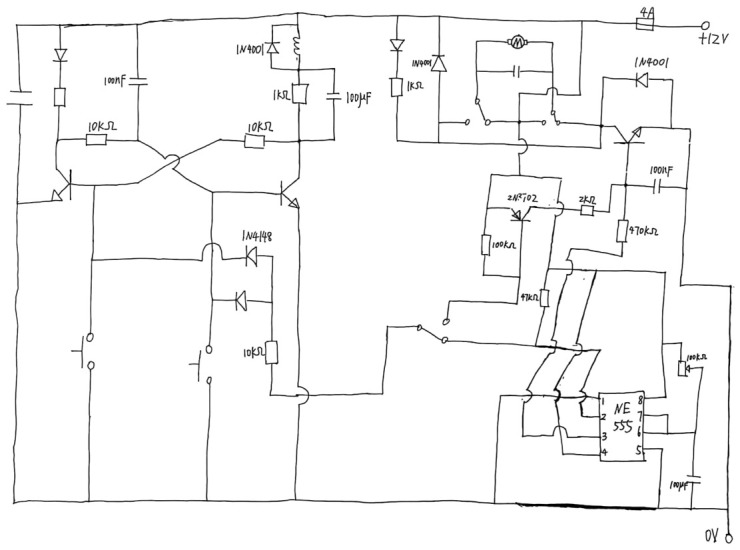
Representative hand-drawn NE555-based pulse-width-modulation (PWM) motor-control circuit used to illustrate downstream executable validation. Numbers and arrows in the image indicate annotated circuit regions and signal or branch directions used for visual explanation.

**Figure 9:**
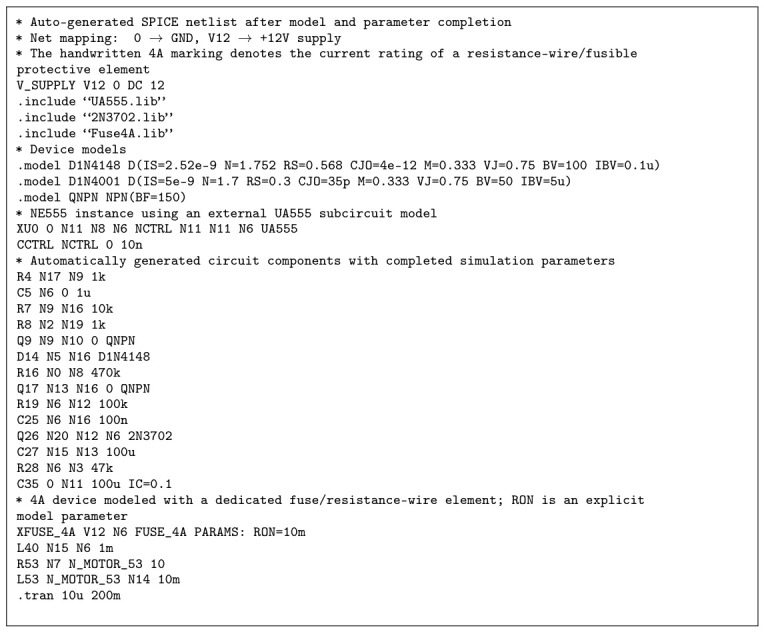
Representative excerpt of the SPICE netlist for the NE555-based PWM motor-control example after model and parameter completion. In this literal SPICE excerpt, lines beginning with “*” are comment lines.

**Figure 10:**
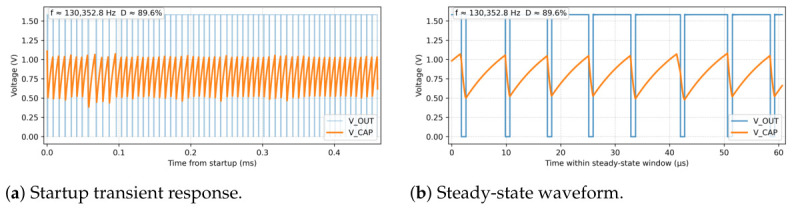
Transient waveforms of the automatically generated NE555 PWM motor-control netlist in ngspice.

To connect the benchmark-level funnel with a concrete circuit instance, we further traced a representative hand-drawn NE555-based PWM motor-control circuit through the downstream validation workflow. Figure 8 shows the original input diagram, Figure 9 shows a representative excerpt of the generated SPICE netlist after simulator-side model and parameter completion, and Figure 10 shows representative transient outputs from the resulting ngspice simulation. In this example, the handwritten “4A” marking in the upper-right branch denotes the rated current of a resistance-wire/fusible protective element rather than a resistance value; it is therefore represented by a dedicated fuse/resistance-wire model instance rather than by a 4 Ω resistor. Some passive-component values are not explicitly visible in the hand-drawn figure; for this representative simulation case, these indispensable values were completed during simulator-side model and parameter preparation and are explicitly listed in the SPICE netlist excerpt. Components whose required resistance or capacitance values are absent and cannot be reliably completed are not treated as directly simulation-ready. This example illustrates how a realistically acquired hand-drawn circuit can be parsed into a simulator-readable representation containing sources, device models, subcircuit instantiation, component instances with explicit parameter values, and transient-analysis directives, and how the resulting execution output can be checked against the intended circuit behavior.

Taken together, the benchmark-level funnel and the representative NE555 case show that the recovered outputs are not only structurally interpretable at the image level, but also usable in a downstream SPICE validation workflow that separates netlist exportability, simulator executability, and result-level correctness.

### 3.8. Runtime Characteristics and Complexity Consistency

Table 10 reports the average per-image runtime of each stage up to generation of the machine-readable structured representation, together with the dominant operation type and expected scaling behavior. Since circuit simulation is treated as a downstream validation step rather than as part of the core parsing pipeline, simulator time is not included.

Let P=H×W denote the number of pixels after image resizing, *D* the number of detected components and text regions, *T* the number of OCR crops, *V* the number of stabilized circuit nodes and terminal endpoints, *K* the number of wire connected components, $V_{k}$ the number of candidate nodes/endpoints in the *k*-th connected component, and *E* the number of candidate graph edges. In this implementation, images are processed at a fixed inference scale, so *P* is bounded across the benchmark. The practical runtime is therefore governed mainly by the constant factors of the full-image pixel-level stages and the graph-construction stages.

The timing distribution in Table 10 is consistent with this complexity analysis. The two slowest stages are component suppression and wire enhancement (1.012 s) and connectivity reasoning (1.141 s). Together, they account for 2.153 s of the 2.9285 s total runtime, or approximately 73.5% of the structured-parsing time. This dominance is expected because both stages require full-image geometric operations, connected-component analysis, and graph construction, whereas recognition and fusion stages operate on fixed-size network inputs, localized crops, or comparatively small sets of detected components and endpoints. Thus, the measured runtime is not determined only by the number of neural-network modules, but by the combination of pixel-level processing, connected-component labeling, local endpoint snapping, and sparse graph selection. The reported timings correspond to single-image offline inference (batch size = 1, excluding disk input/output and simulator execution) on a platform equipped with one NVIDIA RTX 4090D GPU (24 GB), 15 allocated CPU cores on an Intel Xeon Platinum 8474C platform, and 80 GB RAM.

### 3.9. Subtask-Level Comparisons on Public Benchmarks

To provide external reference points on shared public data, we evaluate aligned subtasks on JUHCCR-v1 and CGHD [21,25,31]. These results are reported at the subtask level and are intended to complement, rather than replace, the stricter end-to-end evaluation on the independent 1317-image benchmark. We include two CGHD detection references. The first follows the CGHD dataset-paper reference setting, whereas the second follows the Bayer et al. validation drafter split, namely drafters 21–22, which is the closest publicly reported modular graph-extraction baseline for handwritten circuit diagram images [25]. Table 11 reports the aligned public-subtask results on JUHCCR-v1 and CGHD.

On JUHCCR-v1, the framework achieves 98.03% component-classification accuracy (33,329/34,000), exceeding the published 91.15% reference by 6.88 percentage points. On CGHD under the dataset-paper reference setting, it achieves mAP@0.5:0.95 = 0.559 and mAP@0.5 = 0.707, compared with the dataset paper’s Faster R-CNN reference of mAP = 52% [21,31]. More importantly for comparison with the closest modular graph-extraction baseline, under the Bayer et al. validation drafter split, namely drafters 21–22, the detector achieves mAP@0.5:0.95 = 0.348 and mAP@0.5 = 0.537, compared with the 0.180 validation-set mAP reported by Bayer et al. for Faster R-CNN ResNet-152 [25]. This CGHD detection-level comparison provides a directly aligned quantitative reference to Bayer et al., while the independent 1317-image benchmark remains the primary evidence for end-to-end topology-consistent parsing.

For graph connectivity, Bayer et al. [25] present graph assembly and rectification qualitatively through a sample application, but do not provide a dataset-level quantitative endpoint for full topology recovery, such as graph-connectivity accuracy, edge-level precision/recall, graph edit distance, or netlist-equivalence metrics. Therefore, a full end-to-end numerical comparison would not be methodologically aligned: the present framework outputs topology-consistent structured circuit representations with text–component association, endpoint-semantic inference, SPICE-compatible netlist generation, and downstream simulation validation, whereas the publicly reported Bayer et al. results provide a directly reportable object-detection endpoint but not matched annotations or metrics for these later stages. Accordingly, we limit the directly matched quantitative comparison to the shared CGHD object-detection subtask, and use the independent 1317-image benchmark for strict end-to-end evaluation of topology-consistent structural parsing.

## 4. Discussion

The results indicate that the main remaining difficulty in hand-drawn circuit parsing is topology recovery rather than local symbol recognition. In the present benchmark, component recognition, text recovery, and endpoint-semantic inference were already sufficiently strong that the dominant residual failures were concentrated in connectivity reconstruction under fragmented strokes, local geometric ambiguity, and drawing noise. The added terminal-pair graph-level evaluation further qualifies this result: although strict image-level connectivity accuracy is 96.89%, the recovered topologies achieve 99.59% terminal-pair precision, 99.80% terminal-pair recall, and 99.70% terminal-pair F1, with a mean connectivity edit count of 0.12 per image. This pattern suggests that, for realistic hand-drawn circuit images, further progress is more likely to come from stronger connectivity reasoning than from marginal gains in already strong local classifiers.

From a *Sensors* perspective, the relevance of this work lies in robust processing of camera-acquired engineering images. The pipeline is designed for hand-drawn circuit photographs affected by illumination variation, blur, shadows, perspective distortion, paper texture, and stroke degradation, and converts these imperfect inputs into machine-readable circuit structures suitable for netlist export and downstream simulation [4,5,6,26,27]. In this sense, the contribution is not only circuit parsing, but also a vision-based sensing and image-understanding framework for structurally recovering engineering diagrams from real captured images [10,11].

Regarding circuit-domain applicability, the present task should be understood as engineering-diagram sensing, structural parsing, and circuit digitization, rather than as a method specific to either strong-current or weak-current circuit design. The visual parsing stage is mainly determined by the supported symbol vocabulary, drawing conventions, stroke quality, OCR/parameter completeness, recoverable connectivity, and endpoint-semantic definitions, rather than directly by the operating power level of the circuit. Therefore, power-electronic circuits, measurement circuits, educational circuits, maintenance sketches, and early-stage design diagrams are within the intended application scope when their component symbols, terminal semantics, handwritten parameters, and required SPICE models are covered by the trained taxonomy and exporter. Conversely, circuits containing unseen symbols, highly specialized integrated modules, unconventional notation, missing indispensable parameters, or unavailable device models require additional labeled samples, terminal-role definitions, and exporter/model-library support before the same level of performance can be expected. Thus, the current results demonstrate applicability to the circuit categories represented in the training data and independent benchmark, and support extensibility to other circuit families, but should not be interpreted as universal coverage of all possible electrical, power-electronic, or measurement-circuit drawings.

### 4.1. Comparison with Existing Methods

The closest modular graph-extraction baseline is compared at the CGHD object-detection subtask level using the Bayer et al. drafter split (Table 12). At the full graph-extraction level, directly matched numerical comparison remains limited because prior studies differ in reconstruction target, circuit-family scope, and reported validation endpoint, and Bayer et al. do not report dataset-level connectivity or graph-equivalence metrics [18,19,22,25]. The discussion below therefore separates the directly comparable detection-level evidence from the broader system-level positioning of end-to-end topology-consistent parsing.

Table 13 summarizes only those capabilities and validation endpoints that are explicitly reported in the cited studies and are relevant to this comparison. Here, “Family restr.” indicates whether a method is tied to a limited circuit family or template regime. “Validation level” distinguishes the strict image-level protocol adopted here from studies whose reported endpoints are not directly matched. “NR” denotes items not explicitly reported, and “NDA” denotes settings that are not directly aligned.

Within this comparison frame, the present study is best understood as extending hand-drawn circuit parsing toward stricter structural and semantic completeness rather than claiming a universal cross-paper rank. A particularly distinctive aspect is the explicit endpoint-semantic layer: to the best of our knowledge, prior publicly reported systems for general hand-drawn circuit parsing have not modeled terminal-role semantics for direction-sensitive and multi-terminal devices as an explicit output within an integrated end-to-end pipeline [18,19,22,23,24]. Relative to earlier systems that were family-restricted, logic-specific, or evaluated under different endpoints [18,19,22,42], the present framework combines broader hand-drawn circuit coverage, connectivity reconstruction, endpoint-semantic completion, netlist generation, and downstream executable validation under a strict image-level criterion.

### 4.2. Study Limitations

Several limitations remain. First, although the residual failures are now concentrated mainly in connectivity recovery, the current pipeline still depends on sufficiently recoverable wire evidence. As indicated by the failure-mode analysis, weak or thin wire traces, crowded nearby keypoints, and incomplete component suppression remained the dominant contributors in the connectivity-error subset, especially when multiple factors co-occurred in the same image. This means that topology recovery under severely degraded local evidence is still the main unresolved challenge. In addition, the current connectivity module uses interpretable wire-CC constraints and geometric graph selection rather than a learned edge-scoring model. This choice improves controllability and physical interpretability, but a GNN-based edge scorer or graph-refinement module could further improve ambiguous cases when enough graph-level training labels become available.

Second, although the system supports a broad set of component categories, its generalization to unseen component types or drawing conventions outside the current training distribution remains limited. Third, endpoint semantic inference is currently implemented for the direction-sensitive and multi-terminal components covered in the present benchmark, rather than for all possible symbol families. Finally, the downstream simulation funnel was designed to separate parser-side structured-output completeness, simulator-side executability, and result-level correctness. Simulator execution can additionally depend on external device libraries, indispensable passive-component values, active-device supply definitions, device or subcircuit models, and analysis commands that may not be fully specified in the source diagram. Therefore, the reported downstream simulation results should be interpreted as simulator-side validation of recognized and completed SPICE representations, rather than as an unconditional guarantee that every arbitrary hand-drawn circuit can be fully and automatically behavior-verified without additional model, parameter, supply, or analysis-command information.

## 5. Conclusions

This study presents a vision-based framework for topology-consistent structural parsing of hand-drawn circuit diagrams. Rather than treating the task as symbol recognition alone, the framework combines multi-source visual perception, wire connected-component-guided connectivity reasoning, subtype-aware endpoint semantic recovery, and structured fusion to recover electrically meaningful circuit graphs. On a strictly independent 1317-image benchmark, it achieves a 95.14% strict image-level end-to-end success rate and 96.89% connectivity accuracy, while also supporting SPICE netlist generation and downstream ngspice validation. The results indicate that, under realistic hand-drawn variability, connectivity-centered topology recovery remains the decisive bottleneck and that explicitly constrained structural reasoning is essential for robust circuit digitization. Beyond schematic parsing itself, the study supports a vision-based sensing and imaging workflow in which camera-acquired hand-drawn circuit images are processed into machine-readable and simulation-ready circuit representations under realistic acquisition degradation.

## Figures and Tables

**Table 1:** Task-Specific Training Data Sources and Final Evaluation Benchmark.

Data Source	Pipeline Role	Scale/Coverage
CGHD + Digitize-HCD + additional annotated samples from this study	Multi-class detection of components, text, terminals, and crossovers	4548 images
Text crops derived from the detection dataset	Sequence-based OCR training for labels and parameter strings	95,544 cropped text images
Manually annotated hand-drawn circuit images separate from the independent benchmark	Holistically-Attracted Wireframe Parsing (HAWP)-based prediction of wire-junction and component-terminal keypoints	1314 images
Digitize-HCD-derived terminal-semantic samples, manually verified and corrected	Endpoint semantic recognition for direction-sensitive and multi-terminal components	Diodes, BJTs, MOSFETs, DC sources, and operational amplifiers
Independent end-to-end benchmark constructed in this study	Strict final evaluation only; excluded from training, validation, checkpoint selection, and threshold tuning	1317 images

**Table 2:** Independent End-to-End Benchmark Composition.

Item	Value
Total number of test images	1317
Photographs of pre-existing hand-drawn diagrams	972/1317 (73.80%)
Photographs of newly drawn researcher-created diagrams	345/1317 (26.20%)
Images with text annotations (OCR/association applicable)	1278/1317 (97.04%)
Images with endpoint-semantic-sensitive components	1261/1317 (95.75%)
Images containing crossover structures	941/1317 (71.45%)
Component-count strata used for subgroup analysis	Low: 459; Medium: 442; High: 416
Per-image component count distribution (median [IQR], range)	18 [14, 33], 6–53
Per-image text-annotation count distribution (median [IQR], range; applicable images only)	13.5 [10, 20], 5–33

*Note:* Component-count strata are defined by annotated component count per image as low (6–15), medium (16–29), and high (30–53).

**Table 3:** Implementation Environment and Reproducibility Summary.

Item	Specification
Code repository snapshot	GitHub repository: https://github.com/wanghaoyu6661/Hand-Drawn-Circuit-Diagram-Recognition (accessed on 25 May 2026); manuscript snapshot commit 5e5a957
Pretrained weight release	Hugging Face repository: https://huggingface.co/why0722/hcd-circuit-weights (accessed on 24 May 2026); revision 64cb8ab; archive checksums listed in SHA256SUMS.txt
Primary execution environment	Ubuntu Linux; Python 3.9; PyTorch 2.0.1; CUDA 11.8
Circuit simulator	ngspice v45.2
Released environment specification	environment.yml
Representative inference scripts	run_all.sh, src/pipeline/build_connections.py, src/pipeline/build_final_json.py, src/pipeline/build_spice_netlists.py
Protocol-specific evaluation script	scripts/eval/evaluate_dimensions.py
Sample inputs and labels	data/inputs/ and data/ground_truth/

**Table 4:** Headline End-to-End and Dimension-Wise Results on the Independent Test Set.

Item	x/N	Rate (%)	95% Confidence Interval (CI) (%)
Total number of test images	1317	—	—
All criteria satisfied (strict image-level end-to-end success)	1253/1317	95.14	93.84–96.18
Correct component classification	1302/1317	98.86	98.13–99.31
Correct connectivity inference	1276/1317	96.89	95.80–97.70
Correct text recognition and association (headline, all images)	1294/1317	98.25	97.39–98.83
Correct endpoint semantic recognition (headline, all images)	1309/1317	99.39	98.81–99.69

*Note:* Headline metrics are reported on the all-image denominator under the primary strict image-level evaluation protocol. Dashes indicate not applicable entries.

**Table 5:** Graph-Level Connectivity Metrics on the Independent Test Set.

Metric	Result	Interpretation
Image-level connectivity accuracy	1276/1317 = 96.89%	Strict exact-image success
Terminal-pair true positives/ false positives/false negatives	25,158/103/50	Fine-grained connectivity counts
Terminal-pair precision	99.59%	Control of spurious connectivity relations
Terminal-pair recall	99.80%	Recovery of ground-truth connectivity relations
Terminal-pair F1	99.70%	Overall graph-level connectivity quality
Mean connectivity edit count per image	0.12	Average number of pairwise edits per image
Median connectivity edit count per image	0	Typical image-level edit count

*Note:* Connectivity edit count is defined as the number of false-positive plus false-negative terminal-pair connectivity relations. Terminal-pair metrics are aggregated over all test images and are invariant to arbitrary predicted net names.

**Table 6:** Conditional Results on Applicable Image Subsets.

Item	Applicable N	x/N	Rate (95% Confidence Interval (CI))
Conditional text recognition/association accuracy	1278	1255/1278	98.20% (97.31–98.80)
Conditional endpoint semantic accuracy	1261	1253/1261	99.37% (98.75–99.68)

*Note:* Reported only on applicable image subsets.

**Table 7:** Ablation Study for Endpoint Semantic Inference.

Method	Applicable N	x/N	Accuracy
Rule-based orientation mapping	1261	1031/1261	81.76%
Lightweight orientation classifier	1261	1214/1261	96.27%
ViTPose-style endpoint predictor	1261	1253/1261	99.37%

**Table 8:** Recurring contributing factors within the connectivity-error pool.

Observed Factor	Count	Share Within 41 Connectivity-Error Images
Incomplete component suppression	13	31.71%
Weak/thin wire traces	22	53.66%
Crowded nearby keypoints	17	41.46%
Images with multiple contributing factors	8	19.51%

*Note:* Factor counts are non-exclusive. One image may exhibit more than one factor. “Images with multiple contributing factors” is a subset of the above categories and indicates co-occurrence of at least two factors; percentages therefore do not sum to 100%.

**Table 9:** Causes of Initial Non-Executability Among Exported Netlists.

Cause Category	Count	Share
Resolved after supplementing required model libraries	497	497/513 (96.88%)
Still non-simulatable due to missing indispensable handwritten parameters	16	16/513 (3.12%)

*Note:* Percentages are calculated within the initially non-executable exported subset under the baseline simulator environment (n=513).

**Table 10:** Average Per-Image Runtime and Dominant Complexity of the Recognition and Structural Parsing Pipeline.

Processing Stage	Time (s)	Dominant Scaling
Component and text region detection	0.1255	Fixed-scale detector; approximately O(P)
Text recognition and component association	0.08	O(T) crop recognition plus bounded text–component association
Node and endpoint detection	0.371	Fixed-scale wireframe/keypoint inference; approximately O(P)
Node aggregation and endpoint stabilization	0.131	Local spatial consolidation, approximately O(VlogV) with spatial indexing
Component suppression and wire enhancement	1.012	Full-image masking, morphology, and connected-component processing, approximately O(P)
Connectivity reasoning	1.141	Wire-CC labeling, snapping, local edge selection, and bridge validation; approximately O(P+VlogV+ElogE)
Endpoint semantic recognition and fusion	0.068	Supported-device endpoint classification and structured fusion, approximately O(D+V)
Overall structured parsing pipeline	2.9285	Sum of the above stages

**Table 11:** Aligned Public-Subtask Results on JUHCCR-v1 and CGHD.

Dataset	Aligned Public Subtask	Reported Public Anchor	Ours	Difference
JUHCCR-v1	Isolated component classification accuracy on the public benchmark	91.15% accuracy	98.03% (33,329/34,000)	+6.88 pts
CGHD	Component detection on the public benchmark	0.520 mAP	0.559 mAP@0.5:0.95; 0.707 mAP@0.5	Above anchor

**Table 12:** CGHD Object-Detection Comparison under the Bayer et al. [25] Drafter Split.

Method	Detector	Evaluation Subset	Reported Detection Result
Bayer et al. [25]	Faster R-CNN ResNet-152	Validation, drafters 21–22	0.180 reported mAP
Ours	YOLOv10-based detector	Same split	0.348 mAP@0.5:0.95; 0.537 mAP@0.5

*Note:* Bayer et al. report a single validation-set mAP value without an IoU-threshold breakdown. We therefore report our COCO-style mAP@0.5:0.95 as the closest standardized counterpart and additionally provide mAP@0.5 for transparency.

**Table 13:** Comparative positioning under fragmented benchmarks.

Method	Circuit-Family Scope	Family Restr.	Conn.	Endpt. Sem.	Netlist	SPICE	Validation Level
Moetesum et al. [42] (2018)	Limited analog	Yes	No	No	No	No	NDA
Rachala et al. [18] (2022)	Narrower analog circuits	Yes	Yes	NR	Yes	Yes	NDA
Amraee et al. [19] (2022)	Logic-gate diagrams	Yes	Yes	NR	NR	NR	NDA
Bayer et al. [25] (2024)	CGHD handwritten circuit diagrams	No	Yes	NR	NR	NR	Subtask mAP; qualitative graph sample
Bohara et al. [22] (2024)	Power-converter families	Yes	Yes	NR	Yes	Yes	Different eval. endpoint
**Ours**	**Broader hand-drawn circuits with relatively unconstrained topology**	**No**	**Yes**	**Yes**	**Yes**	**Yes**	**Strict image-level**

*Note:* Boldface identifies the proposed method. NR, not explicitly reported; NDA, not directly aligned. For Bayer et al., the quantitative result explicitly reported for direct comparison is object-detection mAP; dataset-level graph/connectivity metrics are not reported.

## Data Availability

The full inference code, configuration files, and example assets supporting this study are publicly available at the project GitHub repository (https://github.com/wanghaoyu6661/Hand-Drawn-Circuit-Diagram-Recognition (accessed on 24 May 2026)), and the pretrained model weights are publicly available at the associated Hugging Face repository (https://huggingface.co/why0722/hcd-circuit-weights (accessed on 24 May 2026)). Public datasets used for module training or aligned subtask evaluation remain available from their original sources under their respective access conditions. The independently constructed 1317-image end-to-end evaluation benchmark has been organized and is planned for public release after publication of this article. To support verification and reproducibility, benchmark metadata, the annotation protocol, and additional materials needed to assess the reported results are available from the corresponding author upon reasonable request.

## References

[1] Davis R. (2007). Magic Paper: Sketch-Understanding Research. Computer.

[2] Tversky B. (2002). What Do Sketches Say about Thinking?. Proceedings of the AAAI Spring Symposium on Sketch Understanding.

[3] Schütze M., Sachse P., Römer A. (2003). Support Value of Sketching in the Design Process. Res. Eng. Des..

[4] Mohsenzadegan K., Tavakkoli V., Kyamakya K. (2022). A Smart Visual Sensing Concept Involving Deep Learning for a Robust Optical Character Recognition under Hard Real-World Conditions. Sensors.

[5] Matsuo Y., Aoki Y. (2024). Synthetic Document Images with Diverse Shadows for Deep Shadow Removal Networks. Sensors.

[6] Michalak H., Okarma K. (2020). Robust Combined Binarization Method of Non-Uniformly Illuminated Document Images for Alphanumerical Character Recognition. Sensors.

[7] Ding B., Teng Y., Huang Z., Wen L., Li C., Jiang L. (2025). DUFA-Net: A Deep Learning-Based Method for Organ-Level Segmentation and Phenotype Extraction of Maize 3D Point Clouds. Agriculture.

[8] Li C., Yang J., Teng Y., Wang Z., Ma S., Wen L., Huang Z., Zhang Y., Liang L., Yao H. (2026). On-demand Design of Multi-high-Q Terahertz Biosensors Based on Multi-neural Network Fusion. Phys. Lett. A.

[9] Li C., Chen H., Zhu Y., Wang T., Teng Y., Liang L., Zhang Y., Yao H., Huang Z., Jiang L. (2025). Multi-high-Q Terahertz Biosensors Based on a Dynamic Multi-objective Optimization Strategy. Opt. Laser Technol..

[10] Bray N., Hempel M., Boeding M., Sharif H. (2026). Decoding Technical Diagrams: A Survey of AI Methods for Image Content Extraction and Understanding. Information.

[11] Ding Y., Han S.C., Lee J., Hovy E. (2026). Deep Learning Based Visually Rich Document Content Understanding: A Survey. Artif. Intell. Rev..

[12] Bayer J., Diem M., Sablatnig R. (2024). Recognition of Hand-Drawn Electrical Circuit Diagrams. Pattern Recognit..

[13] Lladós J., Valveny E., Sánchez G., Martí E. (2002). Symbol Recognition: Current Advances and Perspectives. Graphics Recognition Algorithms and Applications; Lecture Notes in Computer Science.

[14] Feng G., Viard-Gaudin C., Sun Z. (2009). On-Line Hand-Drawn Electric Circuit Diagram Recognition Using 2D Dynamic Programming. Pattern Recognit..

[15] Agrawal V., Jagtap J., Kantipudi M.V.V.P. (2024). An Overview of Hand-Drawn Diagram Recognition Methods and Applications. IEEE Access.

[16] Hines J.D., Hammond T. A Framework for Recognizing Hand Drawn Diagrams Based on Visible and Invisible Strokes. Proceedings of the 18th International Conference on Pattern Recognition (ICPR).

[17] Sezgin M., Stahovich T., Davis R. Sketch Based Interfaces: Early Processing for Sketch Understanding. Proceedings of the Workshop on Perceptive User Interfaces.

[18] Rachala R.R., Panicker M.R. (2022). Hand-Drawn Electrical Circuit Recognition Using Object Detection and Node Recognition. SN Comput. Sci..

[19] Amraee S., Chinipardaz M., Charoosaei M., Mirzaei M.A. (2022). Handwritten Logic Circuits Analysis Using the YOLO Network and a New Boundary Tracking Algorithm. IEEE Access.

[20] Bohara A., Rani A., Ajjampur N.N., Sethi A. AnchorDETR for Handwritten Circuit Component Detection and Classification. Proceedings of the IEEE/CVF Winter Conference on Applications of Computer Vision Workshops (WACVW).

[21] Roy A., Pani S., Malakar S., Cuevas E., Pérez-Cisneros M., Sarkar R. (2025). JUHCCR-v1: A Database for Hand-Drawn Electrical and Electronics Circuit Component Recognition. Sci. Rep..

[22] Bohara B., Krishnamoorthy H.S. (2024). Deep Learning-Based Framework for Power Converter Circuit Identification and Analysis. IEEE Access.

[23] Kelly C.R., Cole J.M. (2024). Digitizing Images of Electrical-Circuit Schematics. APL Mach. Learn..

[24] Hemker D., Maalouly J., Mathis H., Klos R., Ravanan E. (2024). From Schematics to Netlists—Electrical Circuit Analysis Using Deep-Learning Methods. Adv. Radio Sci..

[25] Bayer J., van Waveren L., Dengel A. (2024). Modular Graph Extraction for Handwritten Circuit Diagram Images. arXiv.

[26] Joffe I., Qian Y., Talebi-Kalaleh M., Mei Q. (2024). A Computer Vision Framework for Structural Analysis of Hand-Drawn Engineering Sketches. Sensors.

[27] Wang D., Fan S. (2026). Research on an Automatic Solution Method for Plane Frames Based on Computer Vision. Sensors.

[28] Liu C., Chitnis D. (2025). EEschematic: Multimodal-LLM Based AI Agent for Schematic Generation of Analog Circuit. arXiv.

[29] Dong Z., Cao W., Zhang M., Tao D., Chen Y., Zhang X. CktGNN: Circuit Graph Neural Network for Electronic Design Automation. Proceedings of the 11th International Conference on Learning Representations.

[30] Zhang G., He H., Katabi D. Circuit-GNN: Graph Neural Networks for Distributed Circuit Design. Proceedings of the 36th International Conference on Machine Learning.

[31] Thoma F., Bayer J., Li Y., Dengel A. (2021). A Public Ground-Truth Dataset for Handwritten Circuit Diagram Images. Proceedings of the Document Analysis and Recognition—ICDAR 2021 Workshops; Lecture Notes in Computer Science.

[32] Ahmed N., Adnan M.F., Shafiullah A., Parash H.J., Rahman M.S., Akib I.C., Sarowar G. (2025). Digitize-HCD: A Dataset for Digitization of Handwritten Circuit Diagrams. Data Brief.

[33] Wang A., Chen H., Liu L., Chen K., Lin Z., Han J., Ding G. (2024). YOLOv10: Real-Time End-to-End Object Detection. arXiv.

[34] Bautista D., Atienza R. (2022). Scene Text Recognition with Permuted Autoregressive Sequence Models. Proceedings of the Computer Vision—ECCV.

[35] Xie K., Gao T., Gao W., Huang J. Holistically-Attracted Wireframe Parsing. Proceedings of the IEEE/CVF Conference on Computer Vision and Pattern Recognition (CVPR).

[36] Huang K., Wang Y., Zhou Z., Ding T., Gao S., Ma Y. Learning to Parse Wireframes in Images of Man-Made Environments. Proceedings of the IEEE/CVF Conference on Computer Vision and Pattern Recognition (CVPR).

[37] Ester M., Kriegel H.P., Sander J., Xu X. A Density-Based Algorithm for Discovering Clusters in Large Spatial Databases with Noise. Proceedings of the 2nd International Conference on Knowledge Discovery and Data Mining (KDD).

[38] Xu Y., Zhang J., Zhang Q., Tao D. ViTPose: Simple Vision Transformer Baselines for Human Pose Estimation. Proceedings of the Advances in Neural Information Processing Systems.

[39] Oquab M., Darcet T., Moutakanni T., Vo H., Szafraniec M., Khalidov V., Fernandez P., Haziza D., Massa F., El-Nouby A. (2023). DINOv2: Learning Robust Visual Features without Supervision. arXiv.

[40] The ngspice Development Team Ngspice: Open-Source SPICE Simulator, Version 45.2; The Ngspice Project, 2025. https://ngspice.sourceforge.io/.

[41] Wilson E.B. (1927). Probable Inference, the Law of Succession, and Statistical Inference. J. Am. Stat. Assoc..

[42] Moetesum M., Younus S.W., Warsi M.A., Siddiqi I. (2018). Segmentation and Recognition of Electronic Components in Hand-Drawn Circuit Diagrams. EAI Endorsed Trans. Scalable Inf. Syst..

